# Implementing Plan of the Day for Cervical Cancer: A Comparison of Target Volume Generation Methods

**DOI:** 10.1016/j.adro.2024.101560

**Published:** 2024-07-01

**Authors:** Lei Wang, Jonathan Mohajer, Helen McNair, Emma Harris, Susan Lalondrelle

**Affiliations:** The Joint Department of Physics at the Institute of Cancer Research and the Royal Marsden NHS Foundation Trust, Sutton, Surrey, United Kingdom

## Abstract

**Purpose:**

Owing to substantial interfraction motion in cervical cancer, plan-of-the-day (PotD) adaptive radiation therapy may be of benefit to patients. Implementation is limited by uncertainty over how to generate the planning target volumes (PTVs). We compared published methods on our own patients.

**Methods and Materials:**

Forty patients each had 3 planning scans with variable bladder filling and daily cone beam computed tomographies (cone beam CTs) during radiation therapy; 5 to 11 cone beam CTs were selected to represent interfraction motion. Clinical target volumes (CTVs) and organs at risk were contoured following EMBRACE-II guidelines. A literature search identified 30 adaptive and nonadaptive solutions to PTV generation, which we applied to our patients. PTV sizes and mean coverage of the daily CTV were determined. For 11 patients, the clinically implemented, subjectively edited plan library was also investigated.

**Results:**

Eleven studies assessed 15 PotD strategies against nonadaptive comparators on a median of 14 patients (range, 9-23). Some PotD approaches applied margin recipes to the CTV on each planning scan, some modeled the CTV against bladder volume, and others applied incremental isotropic margins to the CTV with a single planning scan. Generally, coverage improved as PTV size increased. The fixed isotropic margin required to provide 100% coverage of all patients was 44 mm, with a mean PTV size of 3316 cm^3^. The PotD strategy with the best coverage was a 2-plan library formed by modeling the CTV against bladder volume with extrapolation; it provided 98% mean coverage with 1419-cm^3^ mean PTV size. A 3-plan library consisting of the CTV on each planning scan with 10-mm margin provided 96% mean coverage with 1346-cm^3^ mean PTV size. The clinically implemented solution that employed subjective extrapolation had mean 100% coverage and 1282-cm^3^ PTV size on the 11-patient subset. Coverage provided by the best nonadaptive strategies was not statistically superior to the best PotD strategy (*P* = .13), but PTVs were larger (*P* = .02).

**Conclusions:**

We identified a modeled 2-plan method and a simple 3-plan method, both of which provided excellent coverage with small PTVs compared with nonadaptive strategies.

## Introduction

External beam radiation therapy is key to the management of locally advanced cervical cancer. The low-risk clinical target volume (CTV_LR_) is a particularly mobile composite structure, consisting of the gross tumor, cervix, uterus, parametria, and upper vagina.^1^ Planning target volumes (PTVs) must be relatively large to encompass interfraction motion. Larger volumes may lead to greater normal tissue toxicity.^2^ Online adaptive radiation therapy is attractive because it presents an opportunity to reduce the PTV. Interest has grown in “plan of the day” (PotD), also known as “library of plans,” an online adaptive strategy in which multiple plans are produced before treatment based on subranges of anticipated motion, with each PTV smaller than a nonadaptive PTV would be. The most appropriate plan is chosen based on soft tissue imaging on each treatment day, maximizing target coverage while minimizing dose to organs at risk (OARs).

Although published studies on cervical cancer have demonstrated dosimetric advantages with PotD compared with those with nonadaptive radiation therapy,^3^ clinical implementation of PotD has been slow. No evidence-based guidelines exist for creating the subrange PTVs. Published methods vary widely. It is impossible to compare performance between published studies because they report varying dosimetry metrics and differ in other aspects of practice such as prone or supine patient positioning and bladder filling protocol, which would affect the magnitude of interfraction motion.^4^ We present a direct comparison of published PotD methods using imaging data from our own institution.

## Methods and Materials

### Standard of care

Forty patients with cervical cancer were included from a single institution. All had given written consent for their anonymized data to be used for research. All patients were treated using 25 daily fractions of external beam radiation therapy over 5 weeks using volumetric modulated arc therapy, weekly cisplatin chemotherapy, and brachytherapy. At pretreatment, patients emptied their bladder before the first computed tomography (CT) scan (CT-EB), drank 350 mL of water, and waited for 1 hour before their second CT scan (CT-FB). They emptied their bladder and drank another 350 mL of water 30 minutes before a magnetic resonance imaging (MRI) scan, aiming for a half-full bladder. All 3 scans were acquired in the supine treatment position. Before each fraction, patients drank 350 mL of water and waited for 1 hour. Patients were encouraged to empty their bowels but were not routinely given laxatives or enemas. Cone beam computed tomographies (cone beam CTs) were acquired daily for soft tissue verification and plan selection.

Before April 2022, 29 patients were treated with a nonadaptive individualized internal target volume (ITV) approach following the EMBRACE-II guidelines.^5^ After April 2022, 11 patients were treated using PotD. To create the plan library, 3 ITVs were manually contoured using the CTs and MRI, which had varying amounts of bladder filling. Each ITV encompassed a subrange of anticipated CTV_LR_ motion. If the rectal size was larger or smaller than typically seen, an additional subjective margin was added in the anterior or posterior direction. If the planning scans failed to demonstrate an adequate range of motion, then interpretation based on experience was used to model the anticipated motion. Each subrange ITV was given a 5-mm margin to the PTV to account for setup errors. A further “robust” PTV was created from a union of the 3 ITVs, with a 7-mm margin, to be used in case no subrange PTV covered the CTV_LR_ on the day.

### Imaging data and manual contours

For each patient, the 3 planning scans (CT-FB, CT-EB, and MRI) and all cone beam CTs were imported and rigidly registered with reference to the pelvic bones using RayStation, version 12-R (RaySearch Laboratories AB). The CTV_LR_, bladder, rectum, sigmoid, and bowel bag were contoured on each planning scan by a clinical oncologist following the EMBRACE-II protocol.^5^ For each patient, 5 cone beam CTs were selected semirandomly by date, ensuring an approximately even spread throughout 5 weeks of radiation therapy. These 5 cone beam CTs were manually contoured following the same protocol as that for the planning scans, omitting the sigmoid because of difficulty of visualization. After this, all cone beam CTs were visually inspected. If any uncontoured cone beam CT had CTV_LR_, bladder and/or rectal shapes sufficiently different from those on the contoured cone beam CTs, that cone beam CT was also contoured. A total of 239 cone beam CTs were contoured, ranging between 5 and 11 per patient, which captured the variation of motion and deformation of the CTV_LR_ and OARs. The high-risk clinical target volume (CTV_HR_ consisting of the gross tumor and uninvolved cervix) was contoured on the planning MRI and deformably mapped to all other scans using a hybrid deformable image registration with the CTV_LR_ as a controlling region of interest. The mapped CTV_HR_ was extended 10 mm craniocaudally within the CTV_LR_ to account for uncertainty.

### Literature search and simulation of plan libraries

PubMed was searched for the terms “plan of the day,” “plan selection,” “library of plans,” “plan library,” “cervix adaptive radiotherapy,” and “cervix radiotherapy organ motion” to identify articles that described PotD margin recipes. Where these articles assessed PotD against nonadaptive strategies such as population margins and individualized ITVs, the nonadaptive strategies were also assessed on our data set using the published methods, to provide context. Following the methods described in each article, each PTV was recreated using our patients’ planning scans. The elective nodal clinical target volume was contoured according to EMBRACE-II guidelines, a 5-mm isotropic margin was added, and a union was created with each PTV to create each final PTV.

### Plan selection

**Figure 1:**
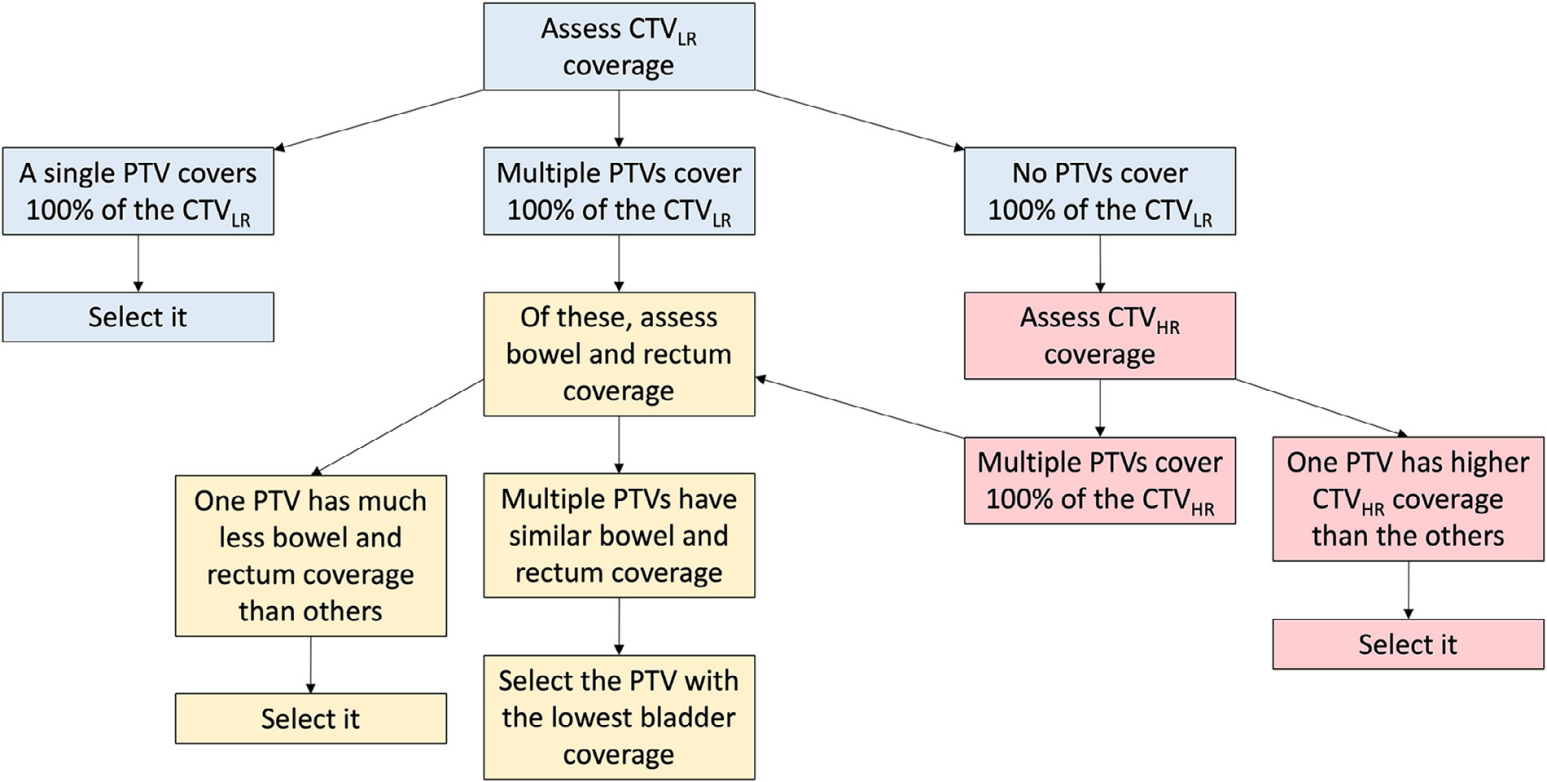
Algorithm for selecting the plan of the day. *Abbreviations:* CTV_LR_ = low-risk clinical target volume; CTV_HR_ = high-risk clinical target volume; PTV = planning target volume.

An in-house script was used within RayStation to calculate the coverage provided by each PTV to each daily CTV_LR_. For PotD approaches, the selected PTV from each library was determined using an algorithm (Fig. 1) based on optimizing coverage of the daily CTV_LR_, CTV_HR_, and OARs. This algorithm is a modified version of our clinically implemented plan selection process without the interventional steps such as patient repositioning. Coverage and volume statistics were recorded for the selected PTV only.

### Statistical analysis

Percentage coverage and PTV sizes were reported as means, medians, SDs, IQRs, and absolute ranges (the latter for coverage only) over all the fractions analyzed. A paired *t* test was used to compare strategies with adjacent mean and median coverage. The published strategies were simulated and assessed on all 40 patients (239 fractions). The subjectively created clinical plan library was only available for 11 patients (61 fractions), so performance was separately compared against the simulated strategies on this subset of patients.

## Results

### Literature search

Eleven articles^6^, ^7^, ^8^, ^9^, ^10^, ^11^, ^12^, ^13^, ^14^, ^15^, ^16^ described their PotD generation in sufficient detail for their methodology to be replicated. These studies either simulated or clinically implemented PotD and compared PotD against other radiation therapy strategies using dose-volume metrics. In total, 39 adaptive and nonadaptive strategies were analyzed in these articles. We excluded 1 strategy^13^ that required a “subjective anisotropic margin,” which would be difficult to reproduce. We excluded 5 PotD strategies that replanned the library during treatment to incorporate CTV_LR_ shapes on cone beam CTs,^14^ which was impractical for our data set. We included the nonadaptive strategies analyzed in the studies to provide useful context for interpreting the results of the PotD strategies. We included the EMBRACE-II fixed margin and individualized ITV approach^5^ because we considered these as the gold-standard nonadaptive approaches. We excluded daily online replanning^6^^,^^15^ or scheduled offline replanning^14^ because these were outside the scope of our study. In total, we assessed 8 population margin approaches, 7 ITV approaches, and 15 PotD approaches (Table 1).

**Table 1:** Adaptive and nonadaptive radiation therapy strategies assessed

Strategy	Published in	Margin recipe
Pop1	Seppenwoolde et al,^10^ 2016	CTV_LR_ on CT-FB + 6 mm
Pop2	Van de Schoot et al,^12^ 2017	CTV_LR_ on CT-FB + 10 mm
Pop3	Ahmad et al,^7^ 2013 O'Reilly and Shaw,^9^ 2016 Seppenwoolde et al,^10^ 2016	CTV_LR_ on CT-FB + 15 mm
Pop4	Bondar et al,^6^ 2012	CTV_LR_ on CT-FB + 16 mm
Pop5	EMBRACE-II protocol^5^	CTV_LR_ on CT-FB + 10 mm (except 5 mm laterally), trim the inferior expansion to match the original CTV_LR_, then + 5 mm
Pop6	Rigaud et al,^14^ 2018	CTV_LR_ on midbladder CT (in this study, we used midbladder MRI) + 7 mm
Pop7	Rigaud et al,^14^ 2018	CTV_LR_ on midbladder CT (in this study, we used midbladder MRI) + 10 mm
Pop8	Bondar et al,^6^ 2012	What is the margin required for adequate coverage of the population?
ITV1	Rigaud et al,^14^ 2018	Union of [CTV_LR_ on CT-EB + CTV_LR_ on CT-FB + CTV_LR_ on midbladder CT (in this study, we used midbladder MRI)] + 7 mm
ITV2	Rigaud et al,^14^ 2018	Union of [CTV_LR_ on CT-EB + CTV_LR_ on CT-FB + CTV_LR_ on midbladder CT (in this study, we used midbladder MRI)] + 10 mm
ITV3	Bondar et al,^6^ 2012	CTV_LR_ motion modeled using linear regression against bladder volume from CT-EB to CT-FB position, extrapolated to bladder volumes of 50 cm^3^ to 1.39 × full bladder volume (capped at 700 cm^3^), + 7 mm
ITV4	Heijkoop et al,^8^ 2014	CTV_LR_ motion modeled using linear regression against bladder volume from CT-EB to CT-FB position, extrapolated to bladder volumes of 50 mL to 1.39 × full bladder volume (capped at 700 cm^3^), + 10 mm
ITV5	Novakova et al, ^11^ 2017	CTV_LR_ motion modeled using linear regression against bladder volume from CT-EB to CT-FB position + 10 mm
ITV6	Van de Schoot et al,^12^ 2017	CTV_LR_ motion modeled using linear regression against bladder volume from CT-EB to CT-FB position + 8 mm (except 13 mm anterior and posterior for the cervix and vagina)
ITV7	EMBRACE-II protocol^5^	CTV_LR_ on CT-FB and CT-EB, with subjective anisotropic expansions to encompass anticipated range of motion, then + 5 mm
PotD1 (2 plans)	Bondar et al,^6^ 2012	CTV_LR_ motion modeled using linear regression against bladder volume from CT-EB to CT-FB position, extrapolated to bladder volumes of 50 cm^3^ to 1.39 × full bladder volume (capped at 700 cm^3^), divided into 2 subranges, + 7 mm
PotD2 (2 plans)	Heijkoop et al,^8^ 2014	CTV_LR_ motion modeled using linear regression against bladder volume from CT-EB to CT-FB position, extrapolated to bladder volumes of 50 cm^3^ to 1.39 × full bladder volume (capped at 700 cm^3^), divided into 2 subranges, + 10 mm
PotD3 (2 plans)	Seppenwoolde et al,^10^ 2016	CTV_LR_ motion modeled using linear regression against bladder volume from CT-EB to CT-FB position, divided into 2 subranges, + 5 mm
PotD4 (2 plans)	Novakova et al,^11^ 2017	CTV_LR_ motion modeled using linear regression against bladder volume from CT-EB to CT-FB position, divided into 2 subranges, + 10 mm
PotD5 (2 plans)	Van de Schoot et al,^12^ 2017	CTV_LR_ motion modeled using linear regression against bladder volume from CT-EB to CT-FB position, divided into 2 subranges, + 8 mm (except 13 mm anterior and posterior for the cervix and vagina)
PotD6 (3 plans)	Zhang et al,^16^ 2022	CTV_LR_ on CT-FB, CT-EB, and midbladder CT (in this study, we used midbladder MRI), no margin
PotD7 (3 plans)	Rigaud et al,^14^ 2018	CTV_LR_ on CT-FB, CT-EB, and midbladder CT (in this study, we used midbladder MRI), + 7 mm each
PotD8 (3 plans)	Rigaud et al,^14^ 2018	CTV_LR_ on CT-FB, CT-EB, and midbladder CT (in this study, we used midbladder MRI), + 10 mm each
PotD9 (3 plans)	Bondar et al,^6^ 2012	CTV_LR_ motion modeled using linear regression against bladder volume from CT-EB to CT-FB position, extrapolated to bladder volumes of 50 cm^3^ to 1.39 × full bladder volume (capped at 700 cm^3^), divided into 3 subranges, + 7 mm
PotD10 (3 plans)	Novakova et al,^11^ 2017	CTV_LR_ motion modeled using linear regression against bladder volume from CT-EB to CT-FB position, divided into 3 subranges, + 10 mm
PotD11 (3 plans)	Van de Schoot et al,^12^ 2017	CTV_LR_ motion modeled using linear regression against bladder volume from CT-EB to CT-FB position, divided into 3 subranges, + 8 mm (except 13 mm anterior and posterior for the cervix and vagina)
PotD12 (4 plans)	Novakova et al,^11^ 2017	CTV_LR_ motion modeled using linear regression against bladder volume from CT-EB to CT-FB position, divided into 4 subranges, + 10 mm
PotD13 (variable no. of plans)	Visser et al,^15^ 2019	Linear regression against bladder volume is used to create discrete simulated CTV_LR_ shapes, + 5 mm (except 10 mm anterior and posterior), trim the inferior expansion to match the original CTV_LR_, then + 5 mm. Number of plans determined by distance between surfaces
PotD14 (margin of the day)	Ahmad et al,^7^ 2013	CTV_LR_ on CT-FB + 0, 5, and 10 mm, and thereafter increasing in multiples of 5 mm until adequate coverage achieved
PotD15 (margin of the day)	O'Reilly and Shaw,^9^ 2016	CTV_LR_ on CT-FB + 0, 5, 7, and 10 mm, and thereafter increasing in multiples of 5 mm until adequate coverage achieved

*Abbreviations:* CT = computed tomography; CT-EB = empty-bladder planning computed tomography; CT-FB = full-bladder planning computed tomography; CTV_LR_ = low-risk clinical target volume; ITV = internal target volume; PotD = plan of the day; PTV = planning target volume.

All margins are isotropic unless otherwise stated. The elective nodal CTV is given a 5-mm margin and a union created with each of the PTVs in the table.

The PotD approaches fell into 3 broad categories. PotD6-8 applied margin recipes onto CTV_LR_ contours to create subrange ITVs. PotD1-5 and 9-13 modeled the CTV_LR_ triangle mesh coordinates against bladder volume to simulate intermediate CTV_LR_ shapes and then incorporated them into subrange ITVs. Some modeling strategies involved extrapolating bladder volumes beyond the range seen at planning to account for emptier and fuller bladders during treatment (PotD1, PotD2, and PotD9). PotD14 and 15 applied isotropic margins of different sizes onto the CT-FB CTV_LR_ and selected the smallest PTV providing full coverage to the CTV_LR_ of the day (also known as margin of the day).

### Coverage and volumes

The fixed isotropic margin required to provide 100% coverage of all fractions for all patients in our data set was 44 mm, resulting in a mean PTV size of 3316 cm^3^ (SD, 504 cm^3^). For context, mean PTV size with no expansion applied to the CT-FB CTV_LR_ was 1094 cm^3^ (SD, 250 cm^3^).

**Figure 2:**
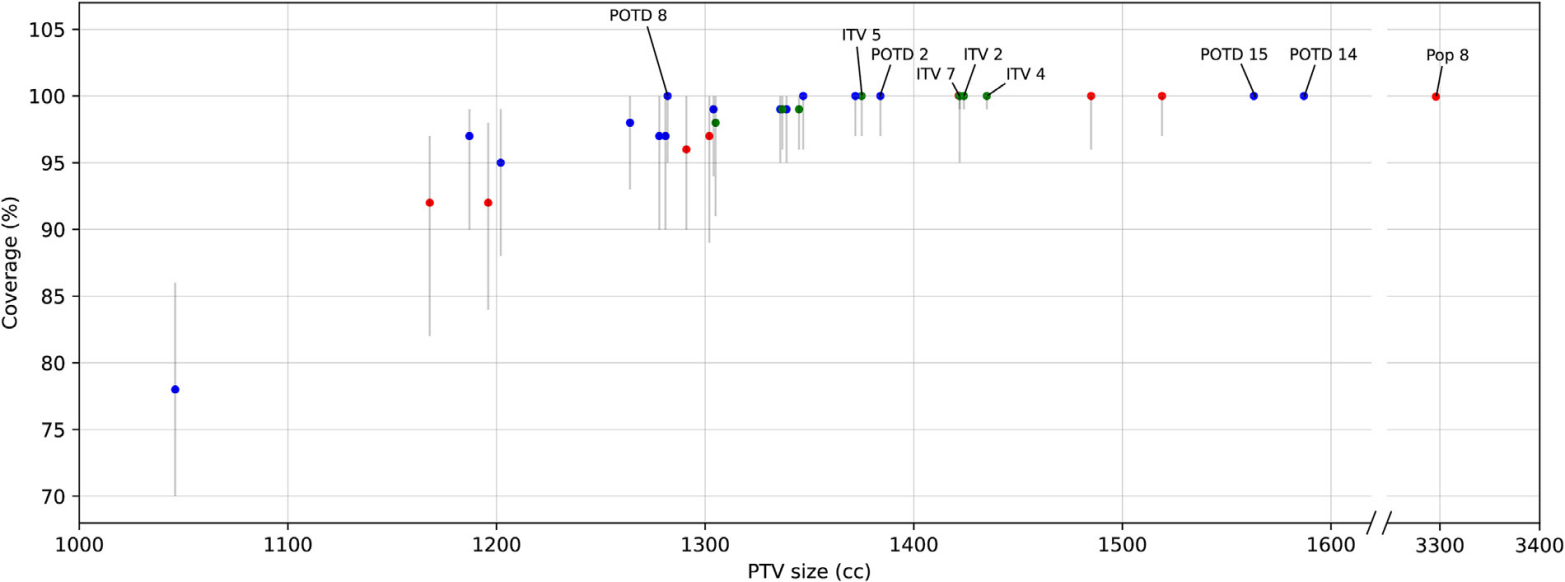
Coverage (%) and planning target volume (PTV) sizes (cm^3^) of simulated strategies on 40 patients. Markers show the median value. Lines show the IQR. For clarity, only strategies discussed in the text have been labeled. See Table 1 for descriptions of PTV names and Table 2 for detailed results. Red, population strategies; green, internal target volume strategies; blue, plan-of-the-day strategies.

For each strategy, results for coverage of the daily CTV_LR_ and size of the PTV (or selected PTV if PotD was used) are shown in Table 2 and Fig. 2. Full coverage in every fraction was achieved by definition in PotD14 and PotD15 (margin of the day) and Pop8 (the 44-mm population margin), with mean PTV sizes of 1698, 1691, and 3316 cm^3^, respectively. PotD2, ITV2, ITV4, ITV5, and ITV7 had the next best coverage, all with mean of 98%, median of 100%, and interquartile range of 97% to 100%. Of these, PotD2 had the smallest mean PTV size at 1419 cm^3^ (compared with mean PTV sizes of 1473, 1501, and 1437 cm^3^, respectively). The coverage of PotD2 and ITV5 was statistically inferior to that of ITV2, ITV4, and ITV 7 (*P* < .01), but the difference was minimal in numerical terms. For the 4 ITV strategies, there was no significant difference in their PTV sizes (*P* = .11). Coverage was not statistically different when comparing PotD2 with the 4 ITV strategies (*P* = .13), but the reduction in PTV size was statistically significant (*P* = .02). Out of all strategies resulting in at least 95% coverage (mean, median, and IQR), the smallest PTV sizes were achieved with PotD8, which had mean PTV size of 1346 cm^3^.

**Table 2:** Coverage (%) and PTV sizes (cm^3^) of simulated strategies on 40 patients, in order of descending mean coverage

Strategy name*	Coverage of daily CTV_LR_ (%)						Fractions fully covered (%)				PTV size (cm^3^)			
	Mean	SD	Median	IQR	Range	*P*†	Covered (%)	All PTVs miss‡	One PTV covers‡	Multiple PTVs cover‡	Mean	SD	Median	IQR
PotD15	100	0	100	100-100	100-100	NA	100				1691	531	1563	1281-1898
PotD14	100	0	100	100-100	100-100	NA	100				1698	527	1587	1325-1898
Pop8	100	0	100	100-100	100-100	NA	100				3316	504	3296	2931-3603
ITV7	98	4	100	99-100	74-100	<.01	67				1458	294	1422	1202-1670
ITV2	98	4	100	99-100	75-100	.98	66				1473	283	1424	1261-1682
ITV4	98	4	100	99-100	76-100	.73	65				1501	309	1435	1289-1632
PotD2	98	4	100	97-100	76-100	<.01	59	41	27	32	1419	288	1384	1207-1566
ITV5	98	4	100	97-100	76-100	.19	54				1437	277	1375	1236-1590
PotD13	97	5	100	97-100	72-100	.17	61	39	13	48	1392	279	1372	1172-1576
Pop4	97	6	100	97-100	59-100	.69	61				1541	297	1519	1297-1716
PotD4	97	5	100	96-100	76-100	.77	51	49	19	32	1387	275	1347	1170-1541
ITV3	97	5	99	96-100	68-100	.60	44				1402	293	1345	1195-1528
ITV1	97	5	99	96-100	67-100	.87	45				1378	273	1337	1164-1587
PotD10	97	5	99	95-100	76-100	.37	49	51	10	39	1370	275	1339	1140-1527
Pop3	97	6	100	96-100	57-100	.88	56				1505	294	1485	1262-1677
PotD12	97	5	99	95-100	76-100	.78	49	51	9	40	1362	275	1336	1130-1520
PotD8	96	7	100	95-100	63-100	.36	51	49	26	25	1346	276	1282	1113-1536
Pop5	96	7	100	95-100	56-100	.93	53				1444	290	1422	1194-1611
PotD1	96	6	99	94-100	65-100	.34	40	60.0	23.0	17.0	1330	276	1304	1102-1467
PotD9	95	7	98	93-100	63-100	<.01	39	61	17	22	1306	273	1264	1091-1447
ITV6	95	7	98	91-100	64-100	.10	34				1339	269	1305	1126-1523
PotD5	94	7	97	90-100	63-100	<.01	33	67	6	27	1320	269	1281	1087-1493
PotD11	94	8	97	90-100	64-100	.06	33	67	3	30	1313	272	1278	1098-1479
PotD7	93	9	97	90-99	57-100	.08	24	76	17	7	1261	268	1187	1044-1439
Pop2	93	9	97	90-100	47-100	.82	31				1344	278	1302	1107-1498
Pop7	93	10	96	90-100	42-100	.51	30				1347	275	1291	1120-1552
PotD3	92	8	95	88-99	55-100	.45	15	85	9	6	1249	260	1202	1039-1381
Pop6	89	13	92	84-98	27-100	<.01	11				1262	266	1196	1053-1484
Pop1	88	12	92	82-97	39-100	.16	7				1234	266	1168	1016-1372
PotD6	76	12	78	70-86	34-97	<.01	0	100	0	0	1097	248	1046	917-1221

*Abbreviations:* CTV_LR_ = low-risk clinical target volume; IQR = interquartile range; ITV = internal target volume; NA = *P* value not applicable because values were identical; PotD = plan of the day; PTV = planning target volume.

⁎ See Table 1 for the description of these contouring strategies.

† Whether coverage is significantly different from the row above, using a paired *t* test.

‡ Not relevant for nonadaptive strategies or margin of the day (PotD14 and PotD15).

For the 11 patients who had prospective PotD treatment, the clinically applied plan library resulted in mean and median coverage of 100% (IQR, 100%; absolute range, 96%-100%). In this subset, PotD14, PotD15, and Pop8 again guaranteed complete coverage with large PTV sizes (mean 1567, 1562, and 3082 cm^3^, respectively). Compared with ITV7, the clinically applied plan library was not statistically different in terms of coverage (*P* = .13) but had a mean PTV size 145 cm^3^ smaller (*P* < .01). All other strategies had statistically inferior coverage to these 2 (*P* < .01). Full results for the 11-patient subset are shown in Table E1 and Fig. E1.

The only difference between PotD14 and PotD15 (margin of the day) was the inclusion of a PTV with a 7-mm margin. In PotD15, the 7-mm margin was used in 21 fractions (8.8%). In PotD14, these fractions were treated with a 10-mm margin instead. PTV sizes increased from mean 1691 cm^3^ with PotD15 (SD, 531 cm^3^) to mean 1698 cm^3^ with PotD14 (SD, 527 cm^3^). The frequency with which each plan was chosen is shown in Figs. E2 and E3.

## Discussion

There is interest in PotD radiation therapy for cervical cancer, but few centers have clinically implemented it.^17^^,^^18^ One major barrier is the lack of guidelines around producing subrange ITVs and PTVs.^3^ Although 11 articles^6^, ^7^, ^8^, ^9^, ^10^, ^11^, ^12^, ^13^, ^14^, ^15^, ^16^ have been published on the benefits of PotD compared with nonadaptive strategies, in terms of improved target coverage or dosimetry and/or improved OAR sparing, it has not been possible to directly compare the performance of their PotD generation strategies owing to differing reporting metrics, patient characteristics, and nonadaptive comparators. The published studies included a median of 14 patients (range, 9-23) and 3 PTV-generation strategies (range, 1-10). In our study, we comprehensively assessed all published and replicable PotD strategies (15 in total; 5 with 2 plans, 6 with 3 plans, 1 with 4 plans, and 3 with a variable number of plans), 13 nonadaptive comparators assessed in those articles, along with the fixed margin and ITV approaches from EMBRACE-II.^5^ We compared 30 PTV-generation strategies head-to-head on the same data set of 40 patients.

PotD2 provided excellent mean and median coverage with narrow IQR and small PTV sizes (Table 2 and Fig. 1). PotD2 is a 2-plan library that is created by contouring the CTV_LR_ on CT-FB and CT-EB, converting the contours to triangle meshes, and modeling the triangle mesh coordinates against bladder volume on CT-FB and CT-EB using linear regression; the model is then extrapolated to larger and smaller bladder sizes and divided into 2 subranges, with a 10-mm margin on each. PotD8 led to even smaller PTV sizes with still excellent coverage. PotD8 is a 3-plan library created by contouring the CTV_LR_ on all 3 planning scans and applying a 10-mm margin on each. PotD2 may be the preferred approach for centers with the capability of reproducing this modeling method, whereas centers that routinely perform 3 planning scans and have capacity for producing 3 plans per library may choose PotD8, which is simpler to replicate and leads to smaller PTV sizes.

The rationale for using PotD is to target radiation therapy to smaller volumes, thereby reducing the dose received by normal tissues, and reducing normal tissue toxicity.^6^ Accordingly, in our data set, PotD did not necessarily provide improved coverage compared with nonadaptive strategies, but the PTV sizes were generally smaller. The strategies with complete coverage of every fraction were Pop8 (an isotropic margin of 44 mm on the CT-FB contour), PotD14, and PotD15 (margin of the day); these nonadaptive strategies ensured complete coverage by definition but resulted in some of the largest PTV sizes.

ITV2, ITV4, ITV5, and ITV7 provided excellent coverage, but PTV sizes were larger on average than those with PotD2 and PotD8. ITV2 is created by a union of CTV_LR_ contours on all 3 planning scans plus a 10-mm margin to PTV. ITV4 and ITV5 employ the modeling approach described above, but without subdivisions: ITV4 extrapolates the range of motion to emptier and fuller bladders, whereas ITV5 encompasses only the range seen at planning. ITV4 had statistically superior coverage to ITV5, but in absolute terms, the difference was minimal. ITV7 is the EMBRACE-II method.^5^ When deciding which strategy to use, centers need to consider the clinical significance of these differences in coverage and PTV size, balanced against the availability of resources for preparing a plan library and online plan selection.

Although studies have shown superiority of PotD compared with nonadaptive cervical radiation therapy,^3^ our results show that the nonadaptive comparator was substandard in some of these studies. For example, Pop1 involves applying a 6-mm margin to the CT-FB CTV_LR_, provided only 88% mean coverage (IQR, 82%-97%) on our data set, and should not be considered a nonadaptive standard of care.

Apart from the strategies that guarantee complete coverage at the cost of large PTV sizes, even the best strategies result in some fractions with poor coverage, as shown by the “Range” column in Table 2. For such fractions, patient repositioning and/or bladder refilling would be necessary before treatment. If the poor coverage is because of a sustained anatomic change such as tumor shrinkage, offline replanning may be appropriate.

At pretreatment, it can be challenging to produce an ITV or subrange ITV that tightly but comprehensively covers a patient's future range of interfraction motion. Ideally, the CT-FB bladder is full, the CT-EB bladder is empty, and the uterocervix is mobile between the 2 scans, providing the clinician with an excellent guide for the potential range of motion. Even in these cases, rectal filling, tumor shrinkage, and other changes may result in poor coverage during radiation therapy. In cases where the CT-FB and CT-EB of bladders are of a similar size, particularly if they are small, or the uterus is immobile at planning despite a good range of bladder volumes, it is particularly difficult to form appropriate ITVs. ITVs limited to the range of motion seen at planning may fall short. Both ITV4 and ITV5 employ the same modeling method with the same ITV-to-PTV margin, except that ITV4 extrapolates beyond the range of bladder volumes seen at planning. Both PotD2 and PotD4 are 2-plan libraries that employ the same modeling method and margins, except that PotD2 extrapolates beyond the range of bladder volumes seen at planning. ITV4 and PotD2 both had statistically superior coverage and covered a greater proportion of fractions compared with ITV5 and PotD4, respectively. However, ITV4 and PotD2 both had larger PTV sizes than their nonextrapolated counterparts.

The plan library clinically applied at our center resulted in excellent coverage and small PTV sizes, appearing to outperform PotD8 and PotD9, but data are only available for the subset of patients who had prospective PotD treatment. The clinical PTVs are roughly based around the CTV_LR_ contoured on CT-EB, CT-FB, and MRI with a 10-mm margin each, but then are substantially edited based on the clinician's subjective assessment of the patient's anatomy. For example, a larger margin is given to the cervix and vagina anteriorly if the rectum is small at planning or posteriorly if the rectum is large at planning; if the bladder volume is limited at planning, the range of CTV_LR_ motion is extrapolated by eye based on estimation of pelvic organ motion. Decisions such as these are challenging to protocolize. There is currently no replacement for clinician experience. Further research will focus on modeling the clinical method and making it widely available for use.

Fully online adaptive radiation therapy involves resegmentation and replanning on the daily scan, achievable using linear accelerators guided by MRI^19^ or high-quality cone beam CT.^20^ As interfraction motion is visualized, these strategies require a smaller margin on the daily CTV_LR_ accounting for setup errors and intrafraction motion. Online replanning has dose-volume advantages compared with PotD and nonadaptive radiation therapy.^6^^,^^15^ However, fully online workflows are more resource intensive for radiation therapy departments and for patients.

Some centers^8^^,^^11^, ^12^, ^13^ have clinically implemented PotD for a subset of patients with cervical cancer designated “movers” based on the distance moved by the tip of the uterine fundus between CT-FB and CT-EB. However, cervical CTV_LR_ motion is complex, and this simple metric is known to result in miscategorizations and the need for replanning.^13^^,^^21^ We have tested all PTV-generation strategies on all patients because we currently lack strong data to inform patient selection.

Our study has several limitations. Contouring on cone beam CT was challenging when the image quality was poor. In particular, the parametria had to be inferred from the shape of the parametria on the planning scans and surrounding anatomy, but this was done with consistent technique and attention. Because interfraction motion of the cervical CTV_LR_ is highly individual, it was considered a priority to include as many unique patients as possible. Although we did not contour every cone beam CT available owing to resource limitation, the methodology used for selecting fractions was designed to capture the extremes of motion for each patient. Compared with randomly selecting 5 fractions per patient, our method enriches for fractions with greater motion. Our results are likely to have underestimated average coverage, which is preferable to overestimating in this context. Because of the large number of patients and strategies assessed, our resources did not allow for planning and dosimetry. PTV size is a surrogate for OAR dosimetry, which is in turn a surrogate for clinical toxicity. PTV size has been found to be positively correlated with toxicity rates in cervix and other pelvic radiation therapies.^2^^,^^22^^,^^23^

## Conclusions

Evaluation of 30 adaptive and nonadaptive PTV-generation strategies demonstrated that coverage generally improved as PTV size increased, although certain PotD strategies punched above their weight. We identified a 2-plan strategy that provided excellent target coverage with small PTV sizes, generated using linear regression modeling of the CTV_LR_ shape against bladder volumes. A 3-plan strategy also had excellent results, generated using the CTV_LR_ shape on 3 planning scans with isotropic margins applied; this may be easier to reproduce, ideal for centers aiming to start PotD treatment.

## Disclosures

Lei Wang reports financial support was provided by Elekta and by Biomedical Research Council, ICR. Jonathan Mohajer reports funding grants from the Cancer Research UK. Helen McNair reports funding grants from National Institute for Health Research and Health Education England. Emma Harris reports funding grants from Elekta and Cancer Research UK. Susan Lalondrelle reports funding grants and speaking and lecture fees from Elekta.

## Appendix Supplementary materials

Fig S1 MotD 13.png Image, image 1

Fig S2 MotD 14.png Image, image 2

Fig S3 MotD 15.png Image, image 3

Supplementary Materials-revised.docx Image, application 4

## Footnotes

Supplementary material associated with this article can be found in the online version at doi:10.1016/j.adro.2024.101560.
