# Supplementary figures and images for: Implementing Plan of the Day for Cervical Cancer: A Comparison of Target Volume Generation Methods
Source: Adv Radiat Oncol. 2024 Jul 1;9(9):101560. doi: 10.1016/j.adro.2024.101560 (PMC11328065; doi:10.1016/j.adro.2024.101560)

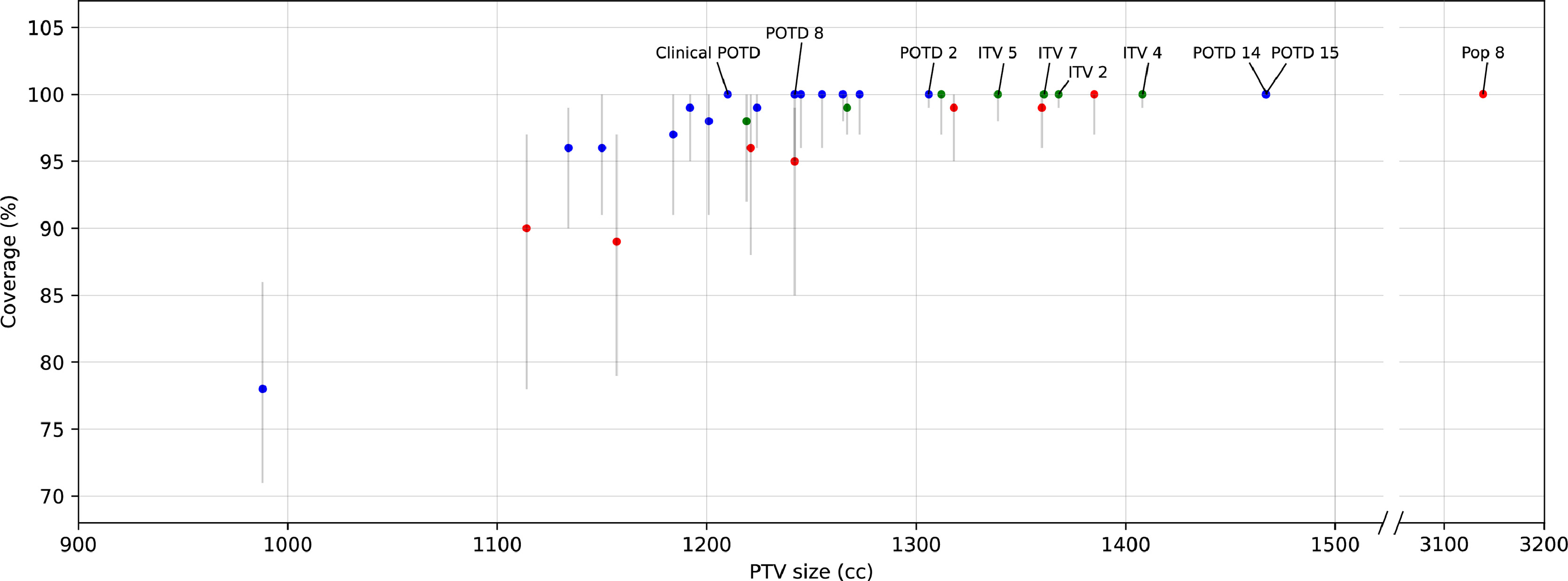

Supplement: Fig S1 MotD 13.png [file mmc1.jpg]

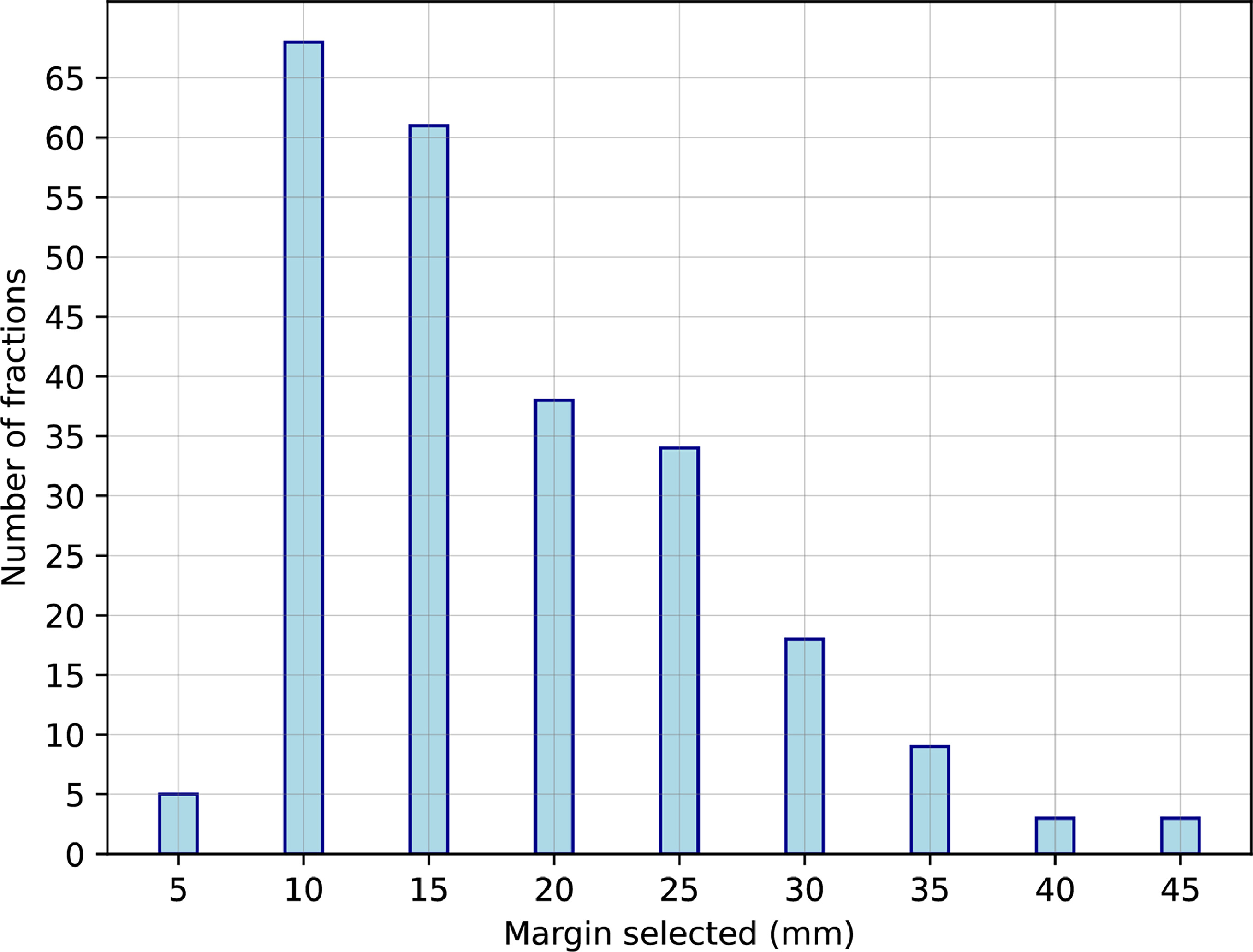

Supplement: Fig S2 MotD 14.png [file mmc2.jpg]

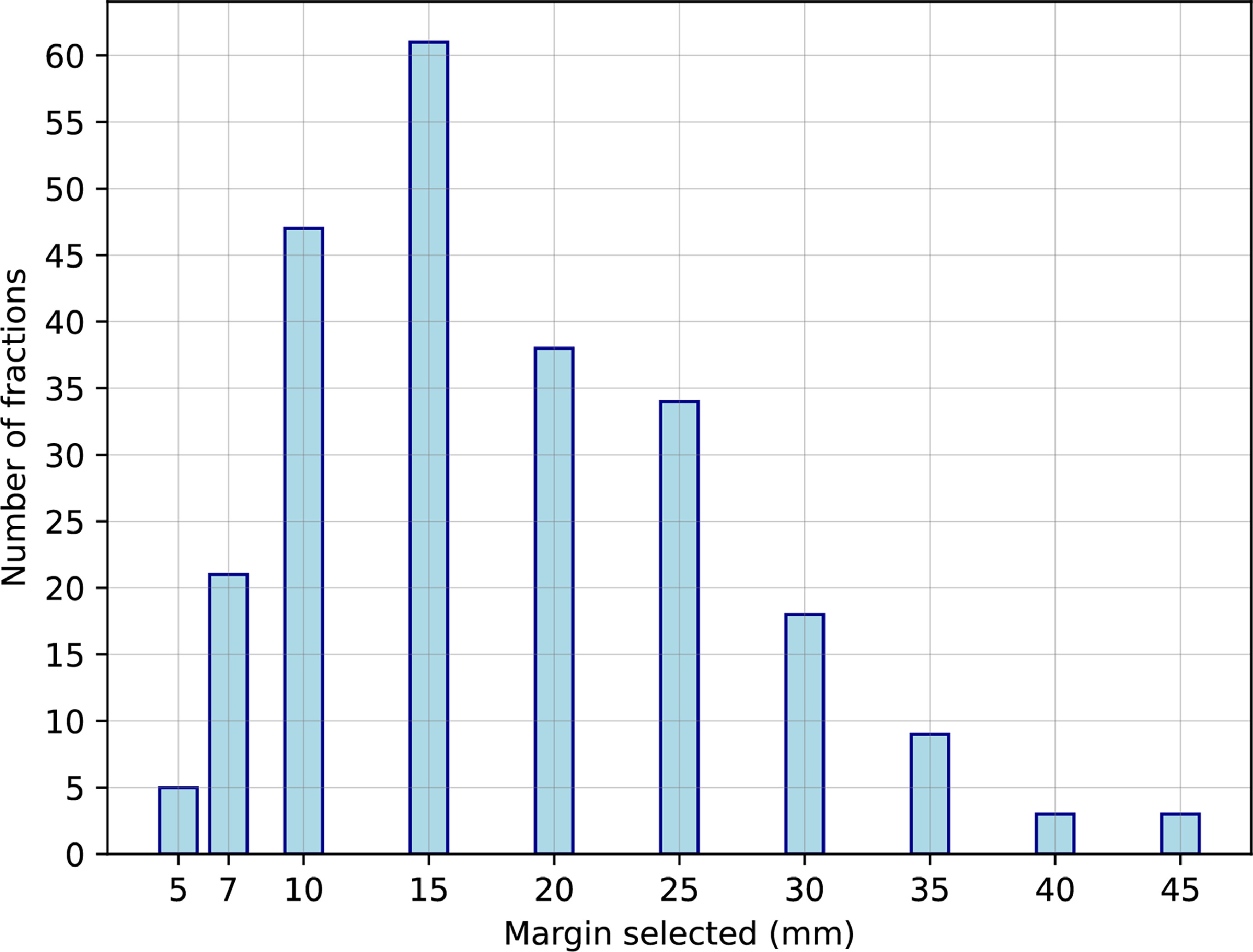

Supplement: Fig S3 MotD 15.png [file mmc3.jpg]
