# Supplementary material for: Implementing Plan of the Day for Cervical Cancer: A Comparison of Target Volume Generation Methods
Source: Adv Radiat Oncol. 2024 Jul 1;9(9):101560. doi: 10.1016/j.adro.2024.101560 (PMC11328065; doi:10.1016/j.adro.2024.101560)

# Supplementary materials

Supplementary Figure 1. Coverage (%) and PTV sizes (cc) for the 11 patients treated clinically with plan-of-the-day. Markers show the median value. Lines show the inter-quartile range. Please see Table 1 for descriptions of PTV names and see Supplementary Table 1 for detailed results. For clarity, only strategies discussed in the text have been labelled. Red; population strategies. Green; internal target volume strategies. Blue; plan-of-the-day strategies.


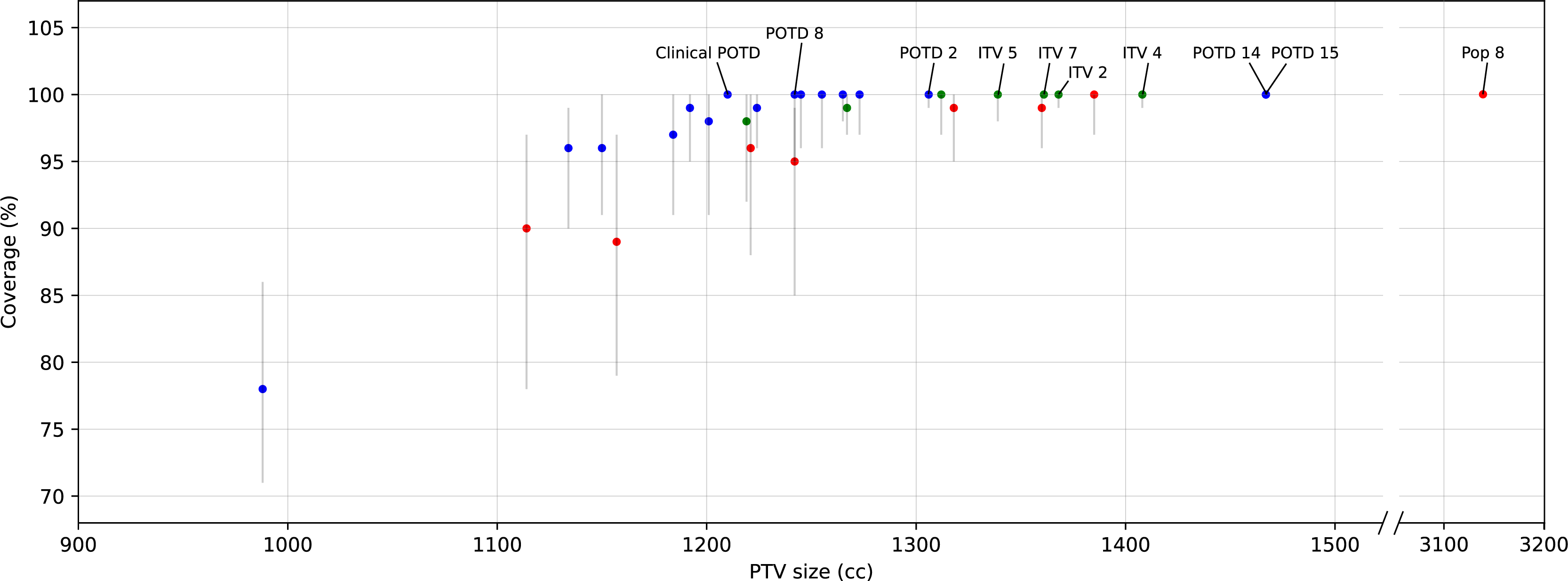


Supplementary Table 1. Coverage (%) and PTV sizes (cc) for the 11 patients treated clinically with plan-of-the-day, in order of descending mean coverage. CTV_LR_; low-risk clinical target volume. SD; standard deviation. IQR; inter-quartile range. NA; p-value not applicable as values were identical.

|  | Coverage of daily CTV_LR_ (%) | | | | | Fractions fully covered (%) | | | | | PTV size (cc) | | | |
| --- | --- | --- | --- | --- | --- | --- | --- | --- | --- | --- | --- | --- | --- | --- |
| Strategy name^a^ | Mean | SD | Median | IQR | Range | p-value^b^ | Covered % | All PTVs miss^c^ | One PTV covers^c^ | Multiple PTVs cover^c^ | Mean | SD | Median | IQR |
| POTD 15 | 100 | 0 | 100 | 100 - 100 | 100 - 100 | NA | 100 |  |  |  | 1562 | 526 | 1467 | 1227 - 1703 |
| POTD 14 | 100 | 0 | 100 | 100 - 100 | 100 - 100 | NA | 100 |  |  |  | 1567 | 522 | 1467 | 1227 - 1703 |
| Pop 8 | 100 | 0 | 100 | 100 - 100 | 100 - 100 | NA | 100 |  |  |  | 3082 | 405 | 3139 | 2778 - 3449 |
| ITV 7 | 100 | 0 | 100 | 100 - 100 | 97 - 100 | 0.02 | 89 |  |  |  | 1427 | 239 | 1361 | 1180 - 1555 |
| Clinical POTD | 100 | 1 | 100 | 100 - 100 | 96 - 100 | 0.13 | 90 | 10.0 | 16.0 | 74.0 | 1282 | 232 | 1210 | 1098 - 1335 |
| ITV 4 | 99 | 2 | 100 | 99 - 100 | 92 - 100 | 0.02 | 72 |  |  |  | 1368 | 189 | 1408 | 1223 - 1536 |
| ITV 2 | 99 | 2 | 100 | 99 - 100 | 90 - 100 | 0.51 | 69 |  |  |  | 1347 | 196 | 1368 | 1209 - 1490 |
| POTD 2 | 99 | 3 | 100 | 99 - 100 | 87 - 100 | 0.23 | 69 | 31 | 44 | 25 | 1282 | 174 | 1306 | 1164 - 1426 |
| ITV 5 | 98 | 3 | 100 | 98 - 100 | 90 - 100 | 0.59 | 57 |  |  |  | 1306 | 178 | 1339 | 1166 - 1468 |
| POTD 4 | 98 | 4 | 100 | 97 - 100 | 83 - 100 | 0.03 | 57 | 43 | 31 | 26 | 1248 | 170 | 1273 | 1131 - 1393 |
| ITV 3 | 98 | 3 | 100 | 97 - 100 | 87 - 100 | 0.96 | 51 |  |  |  | 1275 | 175 | 1312 | 1144 - 1417 |
| POTD 13 | 98 | 6 | 100 | 98 - 100 | 74 - 100 | 0.61 | 66 | 34 | 13 | 53 | 1239 | 172 | 1265 | 1125 - 1372 |
| ITV 1 | 98 | 4 | 99 | 97 - 100 | 85 - 100 | 0.94 | 44 |  |  |  | 1256 | 186 | 1267 | 1137 - 1377 |
| POTD 10 | 97 | 5 | 100 | 96 - 100 | 79 - 100 | 0.54 | 54 | 46 | 20 | 34 | 1228 | 168 | 1255 | 1120 - 1368 |
| POTD 12 | 97 | 5 | 100 | 96 - 100 | 76 - 100 | 0.49 | 54 | 46 | 13 | 41 | 1218 | 166 | 1245 | 1114 - 1356 |
| POTD 1 | 97 | 4 | 99 | 96 - 100 | 80 - 100 | 0.93 | 48 | 52 | 33 | 15 | 1198 | 164 | 1224 | 1088 - 1311 |
| POTD 8 | 96 | 7 | 100 | 95 - 100 | 63 - 100 | 0.31 | 52 | 48 | 33 | 19 | 1207 | 182 | 1242 | 1071 - 1319 |
| POTD 9 | 96 | 6 | 99 | 95 - 100 | 72 - 100 | 0.98 | 48 | 52 | 31 | 17 | 1169 | 160 | 1192 | 1071 - 1277 |
| Pop 4 | 96 | 8 | 100 | 97 - 100 | 59 - 100 | 0.97 | 57 |  |  |  | 1379 | 177 | 1385 | 1260 - 1547 |
| Pop 3 | 96 | 9 | 99 | 96 - 100 | 57 - 100 | <0.01 | 49 |  |  |  | 1345 | 173 | 1360 | 1232 - 1513 |
| Pop 5 | 95 | 9 | 99 | 95 - 100 | 56 - 100 | <0.01 | 48 |  |  |  | 1289 | 170 | 1318 | 1178 - 1450 |
| ITV 6 | 95 | 6 | 98 | 92 - 100 | 70 - 100 | 0.89 | 30 |  |  |  | 1202 | 171 | 1219 | 1096 - 1332 |
| POTD 5 | 94 | 7 | 98 | 91 - 100 | 70 - 100 | 0.02 | 28 | 72 | 5 | 23 | 1178 | 166 | 1201 | 1074 - 1307 |
| POTD 11 | 94 | 7 | 97 | 91 - 100 | 68 - 100 | 0.15 | 28 | 72 | 3 | 25 | 1167 | 168 | 1184 | 1042 - 1284 |
| POTD 7 | 93 | 9 | 96 | 91 - 100 | 57 - 100 | 0.60 | 30 | 70 | 28 | 2 | 1124 | 169 | 1150 | 985 - 1215 |
| POTD 3 | 93 | 7 | 96 | 90 - 99 | 69 - 100 | 0.55 | 15 | 85 | 10 | 5 | 1118 | 153 | 1134 | 1024 - 1219 |
| Pop 2 | 92 | 11 | 96 | 88 - 100 | 47 - 100 | 0.48 | 28 |  |  |  | 1195 | 156 | 1221 | 1071 - 1319 |
| Pop 7 | 90 | 13 | 95 | 85 - 99 | 42 - 100 | 0.22 | 25 |  |  |  | 1220 | 203 | 1242 | 1012 - 1328 |
| Pop 1 | 85 | 14 | 90 | 78 - 97 | 39 - 100 | 0.01 | 5 |  |  |  | 1092 | 145 | 1114 | 960 - 1183 |
| Pop 6 | 85 | 15 | 89 | 79 - 97 | 35 - 100 | 0.93 | 11 |  |  |  | 1139 | 190 | 1157 | 936 - 1230 |
| POTD 6 | 77 | 12 | 78 | 71 - 86 | 34 - 97 | <0.01 | 0% | 100 | 0 | 0 | 972 | 141 | 988 | 831 - 1046 |
| ^a^Please see Table 1 for the description of these contouring strategies. ^b^Whether coverage is significantly different from the row above, using a paired t-test. ^c^Not relevant for non-adaptive strategies or margin-of-the-day (POTD14 and POTD15). | | | | | | | | | | | | | | |

Supplementary Figure 2. The number of fractions requiring each margin expansion, using the margin-of-the-day approach with 5 mm margin increments^7^.


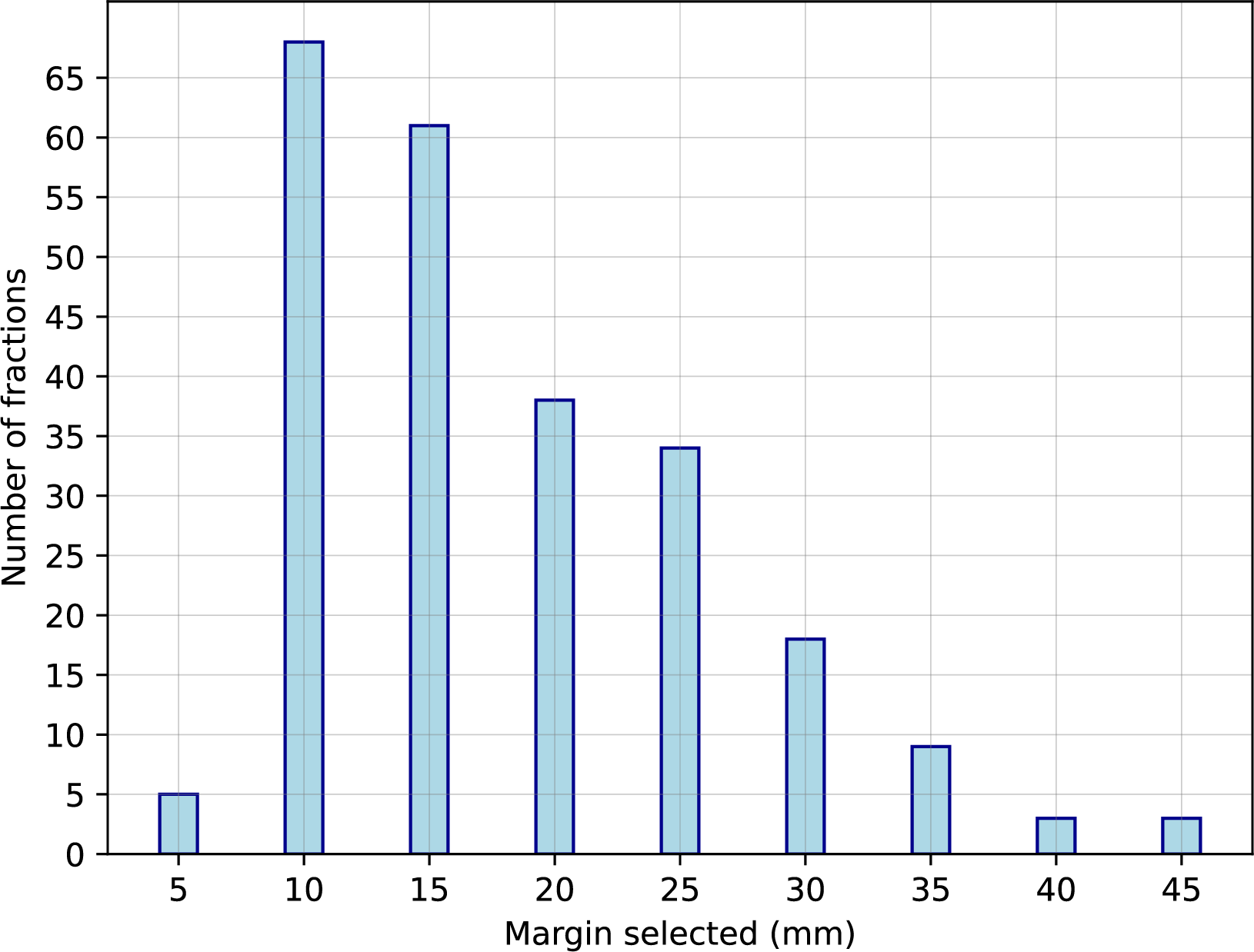


Supplementary Figure 3. The number of fractions requiring each margin expansion, using the margin-of-the-day approach with 5 mm margin increments and a 7 mm margin^9^.


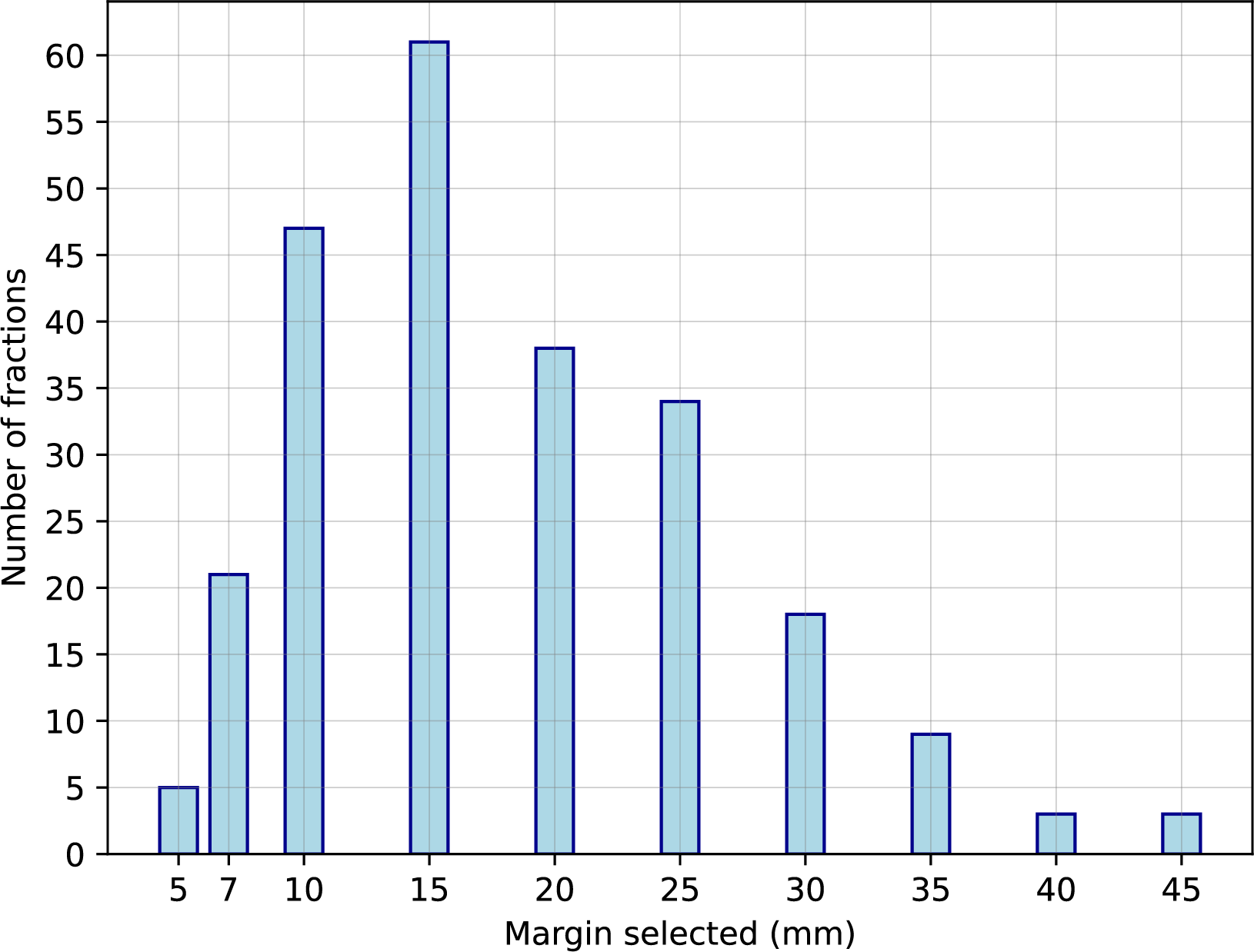

Supplement: Supplementary Materials-revised.docx [file mmc4.docx]
